# Mapping the epidemic changes and risks of hemorrhagic fever with renal syndrome in Shaanxi Province, China, 2005–2016

**DOI:** 10.1038/s41598-017-18819-4

**Published:** 2018-01-15

**Authors:** Weifeng Liang, Xu Gu, Xue Li, Kangjun Zhang, Kejian Wu, Miaomiao Pang, Jianhua Dong, Hunter R. Merrill, Tao Hu, Kun Liu, Zhongjun Shao, Hong Yan

**Affiliations:** 1 Department of Epidemiology and Health Statistics, School of Public Health, Xi’an Jiaotong University College of Medicine, Xi’an, 710061 China; 2 Department of Epidemiology, School of Public Health, Fourth Military Medical University, Xi’an, 710032 China; 3 Department of Epidemiology and Medical Statistics, School of Public Health and Management, Weifang Medical College, Weifang, 261000 China; 4 Department of Mathematics, School of Biomedical Engineering, Fourth Military Medical University, Xi’an, 710032 China; 5 Shaanxi Provincial Corps Hospital of Chinese People’s Armed Police Force, Xi’an, 710054 China; 6 Shaanxi Provincial Center for Disease Control and Prevention, Xi’an, 710054 China; 7 Department of Agricultural and Biological Engineering, University of Florida, Gainesville, Florida 32611 USA; 8 Digital Resources and Information Center, Taishan Medical University, Taian, 271016 China

## Abstract

Hemorrhagic fever with renal syndrome (HFRS) is a major rodent-borne zoonosis. Each year worldwide, 60,000–100,000 HFRS human cases are reported in more than seventy countries with almost 90% these cases occurring in China. Shaanxi Province in China has been among the most seriously affected areas since 1955. During 2009–2013, Shaanxi reported 11,400 human cases, the most of all provinces in China. Furthermore, the epidemiological features of HFRS have changed over time. Using long-term data of HFRS from 2005 to 2016, we carried out this retrospective epidemiological study combining ecological assessment models in Shaanxi. We found the majority of HFRS cases were male farmers who acquired infection in Guanzhong Plain, but the geographic extent of the epidemic has slowly spread northward. The highest age-specific attack rate since 2011 was among people aged 60–74 years, and the percentage of HFRS cases among the elderly increased from 12% in 2005 to 25% in 2016. We highly recommend expanding HFRS vaccination to people older than 60 years to better protect against the disease. Multivariate analysis revealed artificial area, cropland, pig and population density, GDP, and climate conditions (relative humidity, precipitation, and wind speed) as significant risk factors in the distribution of HFRS.

## Introduction

Hemorrhagic fever with renal syndrome (HFRS), a seriously global threat for public health, is caused by Hantaviruses (HVs) in the *Bunyaviridae* family and is characterized by fever, acute renal dysfunction, and hemorrhage manifestations^1–3^. Rodents, shrews, and moles act as the reservoir animals for HVs, and their infection is usually chronic and nearly asymptomatic^4^. Each year worldwide, 60,000–100,000 HFRS cases are reported in more than seventy countries with the majority of these cases occurring in Asia and European developing countries. China accounts for almost 90% of human cases globally^5,6^. In China, Hantaan virus (HTNV) and Seoul virus (SEOV) were identified as the predominant serotypes of HVs which caused human infections. The former is carried by *Apodemus agrarius*, while the latter is associated with *Rattus norvegicus*^6^. Since HFRS was first recognized in northeastern China in 1931, the disease has long been a notable public health problem in China^7^. From 1950 to 2007, approximately 1.56 million HFRS cases have been reported in China, and 46,427 people died from the disease with a fatality rate of 3.0%^6^. The Chinese government paid great attention to anti-HFRS programs, including rodent surveillance and control, public health education, environment management, and especially the free vaccination project^6,8,9^. HFRS incidence has substantively decreased in large parts of China since the year 2000. Nevertheless, HFRS has continuously emerged or reemerged in some locations, and in some epidemic areas HFRS rebounded remarkably^10,11^. Today, HFRS is endemic in all 31 provinces, autonomous regions and municipalities of mainland China, and Hong Kong and Taiwan also found human cases and infected animal reservoirs diagnosed by serology detection^12,13^.

Shaanxi Province, an administrative province in northwestern China, is among the most serious HFRS endemic areas of China since the first case of HFRS was reported in Huxian County in 1955^14^. The highest incidence of HFRS in Shaanxi was recorded in 1984, with an incidence of 38/100,000 persons. Since 2004, the government has supplied a free bivalent vaccine to high-risk people aged 16–60 years in Xi’an Prefecture, the most serious epidemic regions in Shaanxi Province, and gradually expanded the vaccination population^8,15^. Despite this, HFRS incidence in Shaanxi Province is still significantly higher than the average national incidence. According to the Chinese Center for Disease Control and Prevention (CCDC), Shaanxi reported 11,400 HFRS human cases during 2009–2013, the highest of all provinces in China^3,16,17^. Furthermore, characteristics of the epidemic have shifted over time. High endemic areas have spread to peri-urban regions, and localized disease outbreaks occasionally occur^18,19^. For instance, an HFRS outbreak among college students in Xi’an district affected six patients and included two deaths in 2012. In November of the same year, a number of soldiers were infected by hantavirus during field training^20,21^.

As a rodent-borne disease, the occurrences of human infection have been considered a consequence of the distribution and natural history of the reservoir hosts^4^. Human exposure risk is usually determined by the suitability of the environment for HVs, primarily the presence rodent species^1^. Previous studies have shown that the geographical variation of HFRS was associated with ecological factors (e.g., host animals, geographical landscape, topography, climate, etc.) and anthropogenic factors (e.g., agricultural activities, urbanization, and population movements, etc.)^7,10,22–24^. Therefore, a key research priority for effective HFRS prevention and control is improving the knowledge of epidemic characteristics and understanding the underlying risk factors for disease transmission.

Using long-term surveillance data of HFRS from 2005 to 2016, we carried out a retrospective epidemiological study combining geographic information system (GIS) spatial analyses and multivariate ecological models in Shaanxi Province. The aim of this study was to delineate the dynamic epidemic patterns of HFRS, identify the high risk areas, and assess the environmental risk determinants of the disease.

## Results

**Figure 1:**
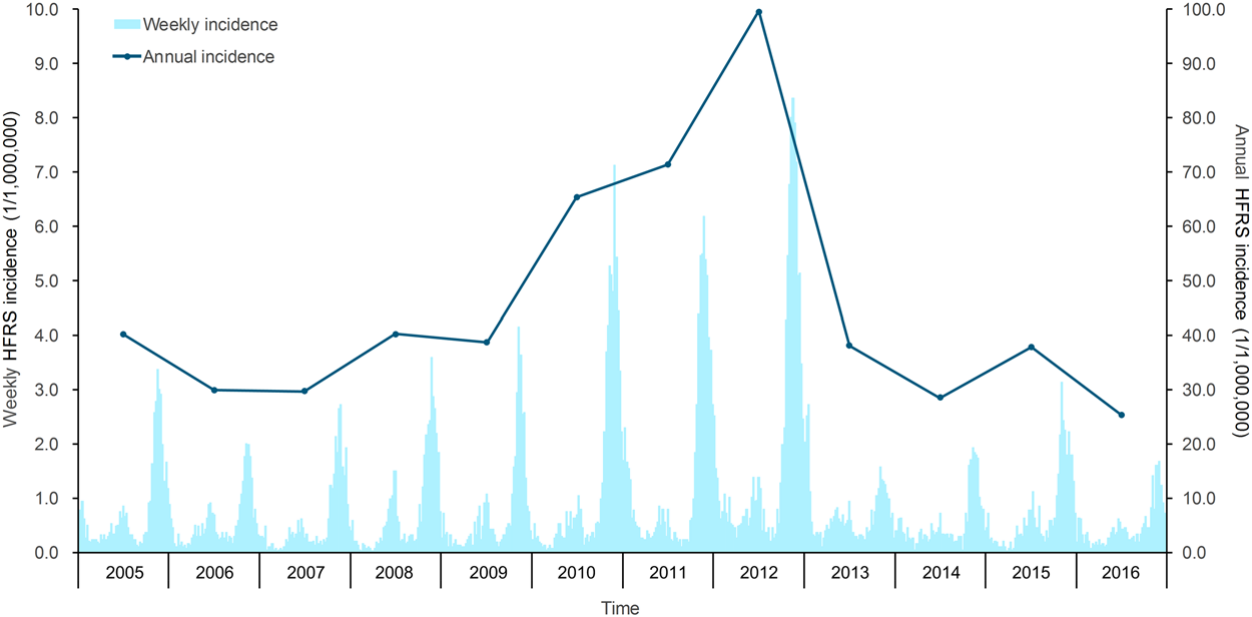
Temporal distribution of the human HFRS incidence in Shaanxi Province, 2005–2016. The bar chart represents the weekly incidence of HFRS, and the line represents the annual incidence of HFRS.

**Figure 2:**
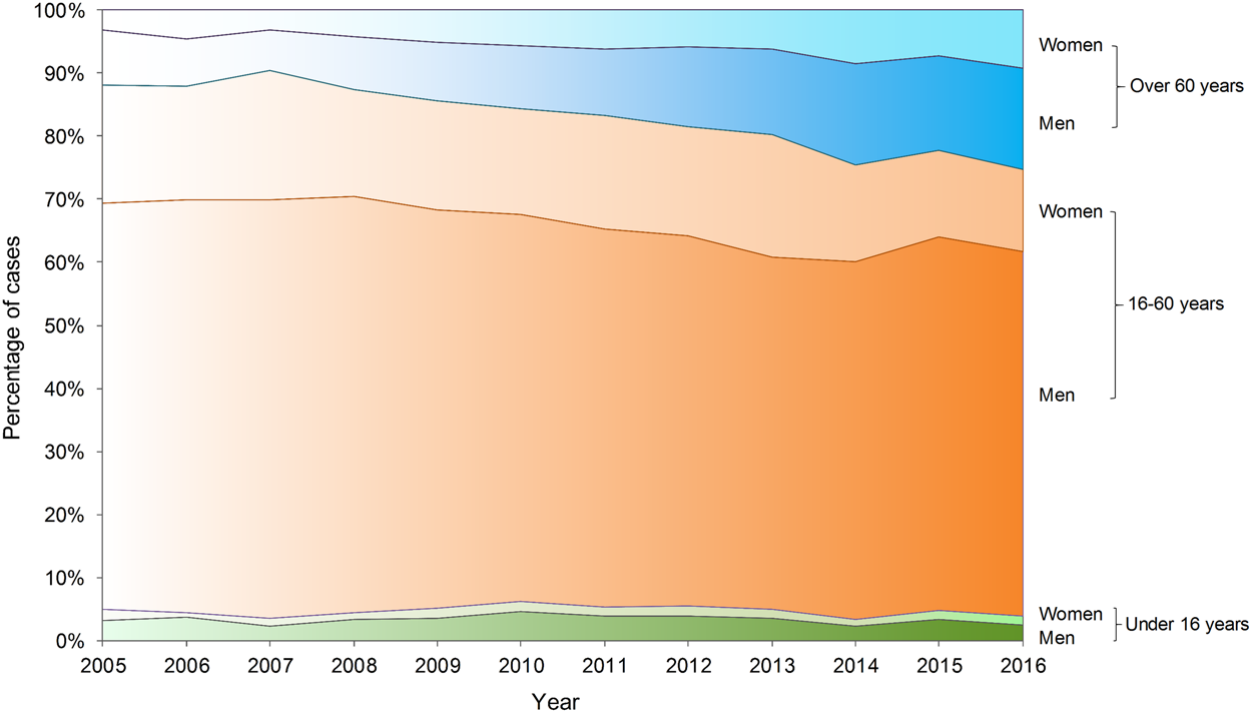
Percentage stacked plot of human HFRS cases by age group and sex in Shaanxi Province, 2005–2016.

A total of 20,142 HFRS human cases and 150 deaths due to HFRS were reported in Shaanxi Province from 2005 to 2016. The annual incidence of HFRS maintained relative stability from 2005 to 2009, and after peaking in 2012 was dramatically reduced from 2012 to 2016, in which the incidence rates per 100,000 persons declined from 9.93 to 2.53. The HFRS weekly incidence revealed an obviously bimodal curve with a major peak from the 43rd week to the 53rd week per year (56.87% of total cases), and a minor peak between the 20th and the 30th week (16.38% of total cases) (Fig. 1). The median age of the cases was 45 years (range, 2–94 years), and the mean (±SD) age was 44 (±16.7). The male-to-female ratio of all cases was 3.10, and males had a significantly higher incidence than females among all age groups (P value < 0.01). In terms of age, although most HFRS cases occurred among persons 16–60 years of age, the proportion decreased from 2005 to 2016, whereas the proportion of cases among those older than 60 years rose by more than 10% (Table 1 and Fig. 2). Figure S1 shows the age groups of men and women aged 60–74 years have become the highest incidence groups since 2011. Figure S2 shows a distinct increase in the annual vaccination population from 2003 to 2013, which may have contributed to a decreasing proportion of HFRS cases among age groups being vaccinated (16–60 years).

**Table 1:** Sex ratio, age group and profession distribution of HFRS patients in Shaanxi Province, China, 2005–2016.

Year	Total cases	Sex ratio (Male: Female)	Age distribution (years)			Profession distribution			
			<16(%)	16–60(%)	>60(%)	Peasant (%)	Student (%)	Retiree (%)	Others (%)
2005	1497	3.19	5.02	83.01	11.97	74.15	9.08	2.54	14.23
2006	1103	3.31	4.56	83.28	12.16	72.89	9.79	2.36	14.96
2007	1091	3.00	3.64	86.58	9.78	73.97	9.26	1.19	15.58
2008	1496	3.51	4.57	82.47	12.96	71.45	10.03	2.21	16.31
2009	1439	3.17	5.08	80.27	14.65	74.15	8.96	2.51	14.38
2010	2434	3.16	6.22	78.02	15.76	72.39	11.26	2.38	13.97
2011	2624	2.89	5.51	77.50	16.99	75.50	9.57	2.28	12.65
2012	3633	3.02	5.70	75.67	18.63	77.32	9.99	2.75	9.94
2013	1416	2.70	5.20	74.59	20.21	75.78	7.84	3.39	12.99
2014	1060	3.02	3.31	71.89	24.80	80.57	6.23	2.74	10.46
2015	1406	3.46	4.93	73.02	22.05	78.52	6.40	3.34	11.74
2016	943	3.21	3.50	71.05	25.45	73.81	6.21	5.2	14.85

**Figure 3:**
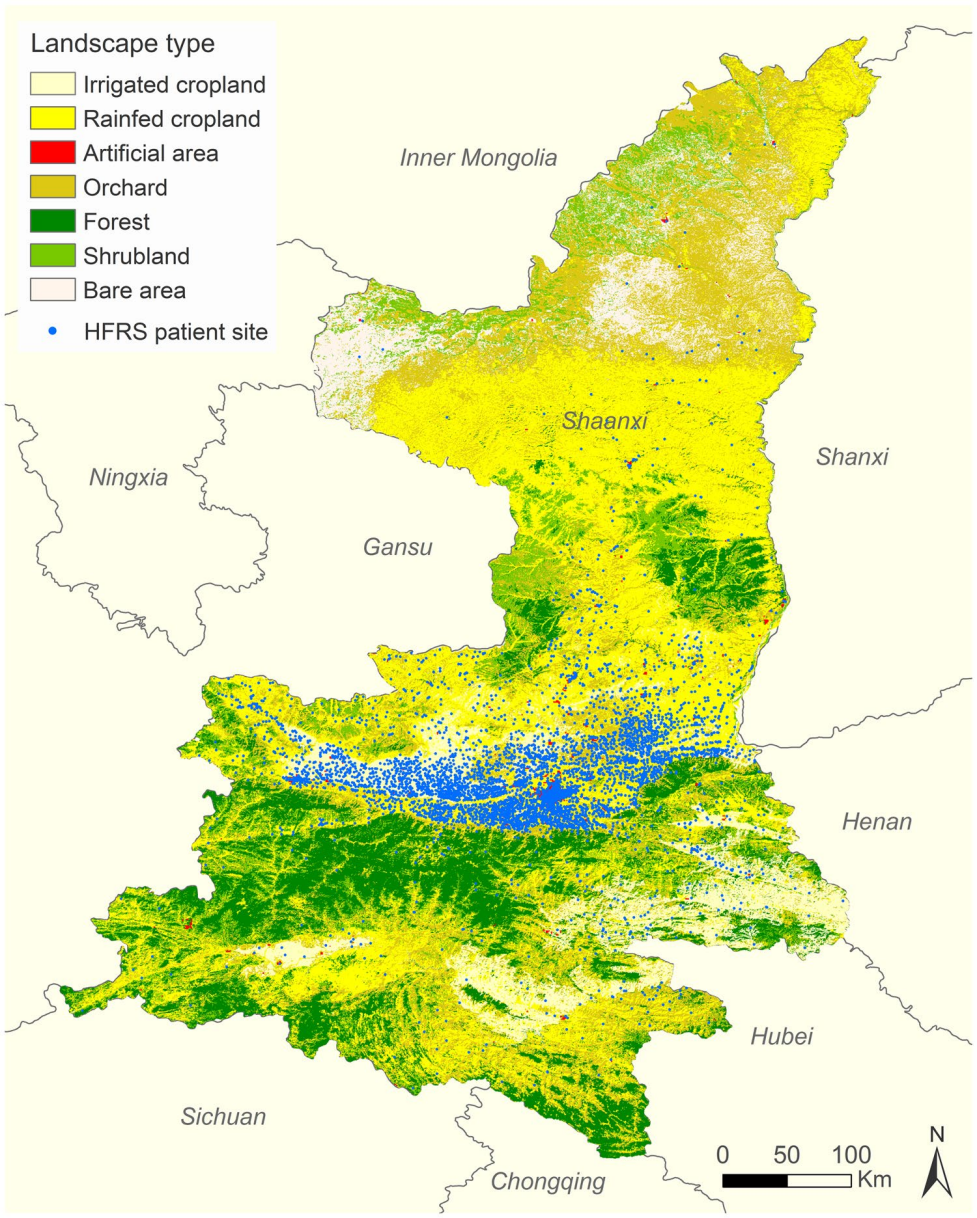
Spatial distribution of the HFRS cases overlapping the map of land cover in Shaanxi Province, 2005–2016. Seven land cover types have been categorized in the study areas: irrigated cropland, rainfed cropland, artificial area, orchard, forest, shrubland, and bare area. The map was created in ArcGIS 9.3 software, ESRI Inc., Redlands, CA, USA, (https://www.arcgis.com/features/index.html).

**Figure 4:**
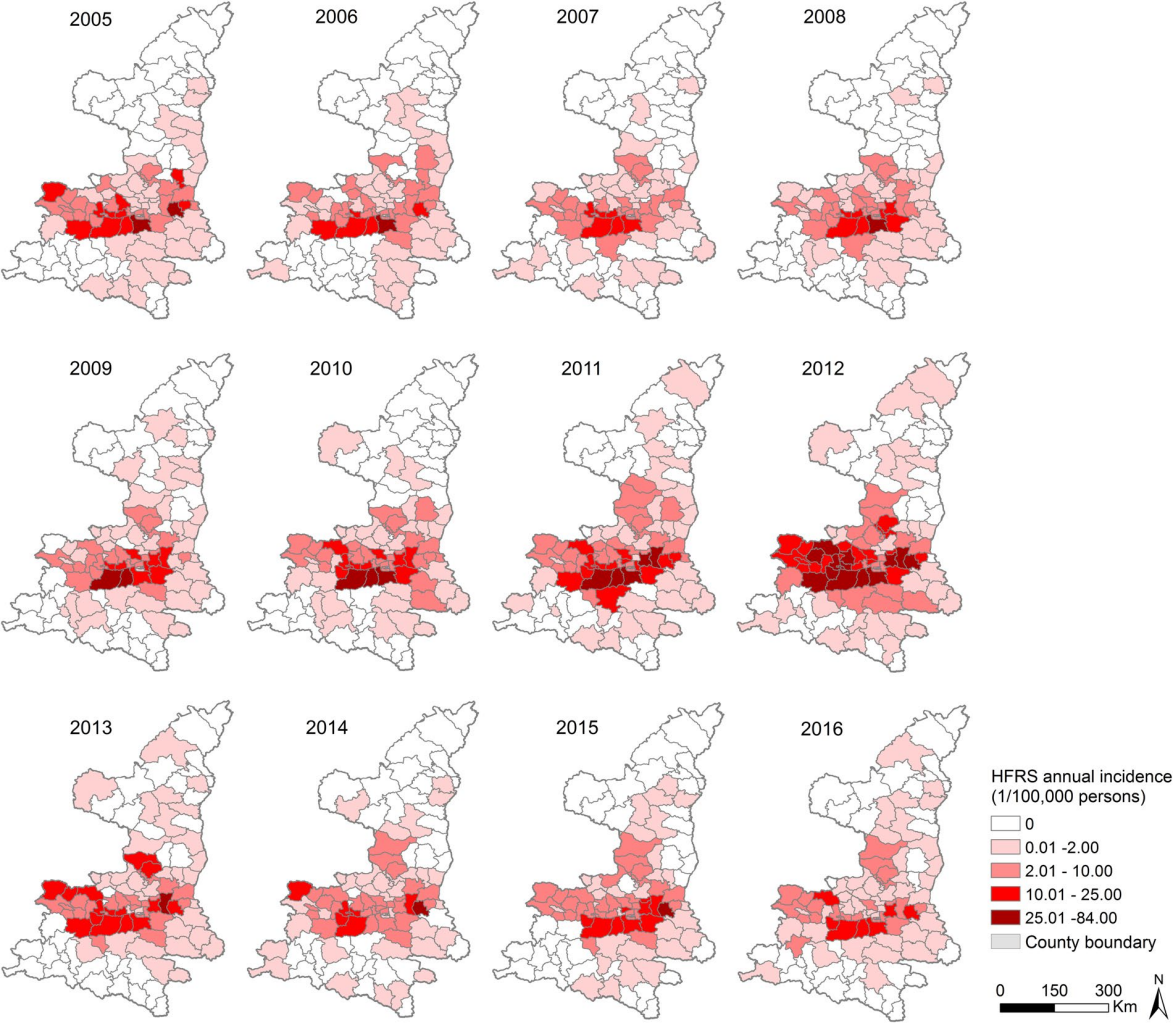
Annual incidence of HFRS for each county in Shaanxi Province, 2005–2016. The map was created in ArcGIS 9.3 software, ESRI Inc., Redlands, CA, USA, (https://www.arcgis.com/features/index.html).

Human HFRS cases were distributed mainly in the Guanzhong Plain in the central parts of Shaanxi Province (Fig. 3), between north latitude 34° and 36°, and rarely in the northern and southern parts (Figure S3). 100 out of 107 counties in Shaanxi Province reported human HFRS cases over the twelve years studied, and the geographic extent of HFRS showed a slowly extending pattern which peaked in 2012 with 82 counties recording HFRS cases (Fig. 4). The five counties with the highest incidences were Chang’an District, Zhouzhi county, Hu county, Wugong county, and Hua county, with the average incidences being 26.49, 23.10, 20.15, 15.11 and 14.83 per 100,000 persons, respectively.

Spatio-temporal scan statistics identified four significant hotspots of HFRS encompassing 33 counties which accounted for 49.55% of the total cases (Figure S4). The relative risk (RR) of HFRS incidence inside the hotspots compared to those outside the hotspots ranged from 3.95 to 10.18. The primary hotspot (hotspot 1) included 10 counties in 5 prefectures (Xi’an, Baoji, Xianyang, Hanzhong, and Ankang), and spanned October 2005 to January 2013 with a RR value of 7.83. Hotspot 2 was situated in 6 counties of Weinan Prefecture with a 3.95 RR value occurring from October 2010 to January 2015. Hotspot 3 was located in mountain areas and loess plateau including 12 counties to the north of the Guanzhong Plain. This hotspot had a RR value of 10.18, and occurred between October and December of 2011. Hotspot 4 was identified in Xi’an central urban area consisting of 5 counties with a 4.81 RR value, and persisted for three months between November 2012 and January 2013 (more detailed information in Table 2 and Figure S4).

**Table 2:** Information for spatio-temporal hotspots of HFRS in Shaanxi Province, China, 2005‒2016.

Hotspots	1	2	3	4
Time period	2005/10-2013/1	2010/10-2015/1	2012/10-2012/12	2012/11-2013/1
No.obs	6766	2620	402	193
No.exp	1222.40	734.22	40.23	40.39
RR	7.83	3.95	10.18	4.81
LLR	6940.52	1542.02	566.86	149.83
Annual incidence	25.1/100,000	16.2/100,000	45.3/100,000	21.7/100,000
No.counties	10	6	12	5
No.population	3,676,149	3,085,279	2,845,946	3,538,789
Area (Km^2^)	15353.55	6041.73	19421.51	756.45
Major geomorphology	Plain and mountainous area	Plain	Hilly area and loess plateau	Urban area
Elevation, median(range)	1091 m (369–3747 m)	604 m (320–2548 m)	1101 m (440–1835 m)	415 m (352–583 m)
Dominant rodents	*A. agrarius*, *M. musculus*	*A. agrarius, R. norvegicus*,	*C. triton, C.barabensis*	*R. norvegicus, M. musculus*
	*R. norvegicus*, *N. niviventer*	*M.musculus*	*A. agrarius, R. norvegicus*	*A. agrarius*

No.obs: number of observed cases; No.exp: number of expected cases;

RR: relative risk for the HFRS incidence in the hotspot compared to the average incidence at the same time period; LLR: log likelihood ratio; No.counties: number of counties within hotspot; No.population: population within the hotspot.

**Figure 5:**
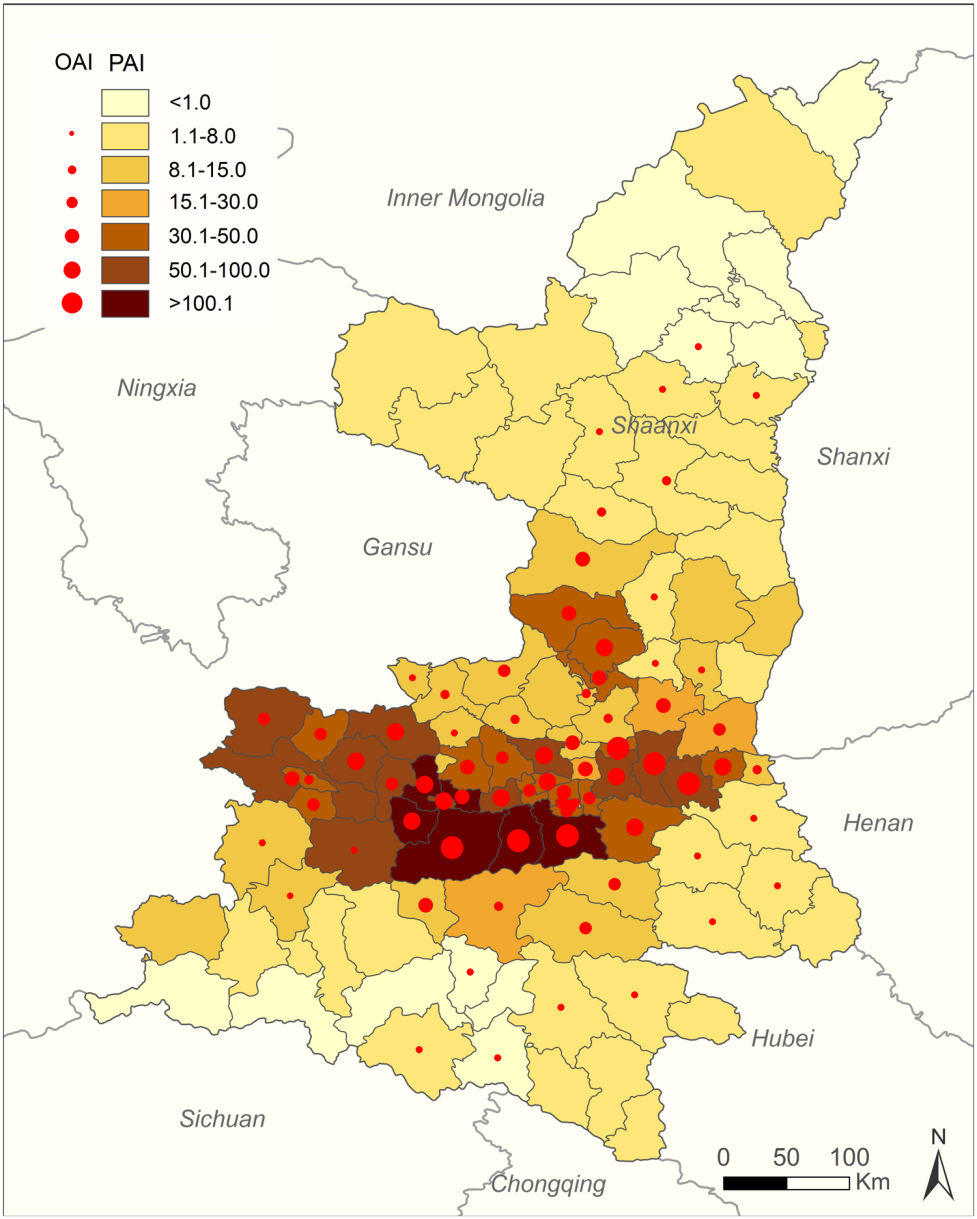
The predicted and observed incidence distribution of HFRS at the county level in Shaanxi Province, 2015–2016. The different color grades represent the predicted incidence of HFRS in 2015–2016 (Unit: 1/1,000,000 person), and red solid circles represent the average observed HFRS incidence in 2015–2016. The map was created in ArcGIS 9.3 software, ESRI Inc., Redlands, CA, USA, (https://www.arcgis.com/features/index.html).

Based on the BRT multivariate model, the geographic distribution of HFRS was found to be significantly associated with eight factors: artificial area, cropland, pig and population density, precipitation, relative humidity, wind speed, and GDP, all with average relative contributions above the threshold of 5.56% (Table 3). The model-fitted partial dependence functions and standard deviations were plotted for each predictor in Figure S5, revealing non-linear relationships between the predictors and HFRS incidence. The predicted incidence of HFRS first increased significantly and then plateaued in response to increase in percentage coverage of artificial area, percentage coverage of cropland, and relative humidity. Similarly, for increasing population density, precipitation, and wind speed, the risk ratios also increased first, then decreased dramatically when passing the peak. Conversely, the HFRS incidence initially decreased and then increased with increasing pig density. A negative relationship was found for GDP, with the highest correlation corresponding to low GDPs of 0~2500 Yuan, then declining for higher values. The pseudo-*R*^2^ statistic suggested that 66.6% of the total geographic variation of HFRS could be explained by the predictive model (Figure S6). The final model was used to map the forecasted geographic distribution of HFRS in 2015–2016 in Fig. 5. The forecasted high endemic areas mainly occur in the central areas of Shaanxi, overlapping well with the 2015–2016 observed incidence.

**Table 3:** Summary of the relative contributions (%) of predictor variables for the HFRS incidence in the boosted regression tree model.

Variable	Boosted regression trees		
	RC(mean)	95%CI	RC(sd)
Temperature	3.68	3.19–4.32	0.28
Precipitation*	7.65	6.92–8.41	0.46
Relative humidity*	7.73	6.51–9.05	0.65
Sunshine hours	2.64	1.28–3.42	0.66
Wind speed*	7.10	6.00–8.56	0.64
Pressure	1.06	0.41–1.51	0.29
Elevation	2.82	2.27–3.22	0.25
Percentage coverage of artificial area*	23.02	21.36–26.60	1.46
Percentage coverage of cropland*	13.21	11.28–14.12	0.75
Percentage coverage of forest	1.48	0.64–1.97	0.36
Percentage coverage of shrub	0.36	0.20–0.53	0.09
Percentage coverage of orchard	0.58	0.27–0.84	0.15
GDP	5.99	5.25–6.71	0.41
Population density*	8.69	7.54–10.22	0.68
Pig density*	9.86	8.49–11.29	0.77
Cattle density	2.82	1.88–3.32	0.41
Goat density	1.22	0.75–1.53	0.22
Selenium	0.11	0.05–0.19	0.04

^*^Variables with relative contribution in the BRT models more than 5.56 were considered to be significantly contributing to the occurrence of human infection with HFRS.

RC: relative contribution.

## Discussion

Since 1955, Shaanxi Province has been among the most seriously HFRS-affected areas in China. To our knowledge, this is the first comprehensive study to delineate the dynamic epidemic changes and risk determinants of HFRS in Shaanxi. The primary results suggested HFRS cases in Shaanxi Province were mainly distributed in the Guanzhong Plain, but the geographic extent of HFRS slowly spread northward over the study period. Profiting from a series of anti-HFRS programs, especially the free HFRS vaccination for people aged 16–60 years, cases in this group have decreased gradually, and the elderly have become the highest-risk population. Artificial area, cropland, pig density, climate, and other ecological factors might provide suitable habitats for rodents or sufficient human exposures, which played important roles in human HFRS occurrences.

Shaanxi Province belongs to the mixed hantavirus epidemic focus where the HTNV serotype caused the major disease burden^14^. Previous surveys revealed that there were at least 50 species of rodents in Shaanxi Province, and *A. agrarius* was the HVs dominant rodent host. Other rodent species including *R. norvegicus*, *Rattus flavipectus*, *Mus musculus*, and *Corcidura suaveolens* could also carry tiny amounts of HVs^15,25^. As a field mice species, *A. agrarius* is widely distributed throughout the Shaanxi Province, especially in Guanzhong Plain which is a famous farming region in the center of Shaanxi (Fig. 3). Most cases were male farmers who lived in rural areas and acquired infection in the winter. Our findings showed a significant change in the age of HFRS cases over the past 12 years, in which the proportion of cases occurring in people older than 60 years has increased by more than 10%, and the highest age-specific attack rate since 2011 occurred among those people aged 60–74 years. We speculate this phenomenon could be explained by the HFRS vaccination program which only focused on people aged 16–60 years, but excluded the elderly^8^. Another likely reason is that many young adults from rural areas migrate to cities for better job opportunities, leaving the elderly and children in the villages. The elderly, in particular elderly males, then have to undertake agricultural activities, exposing themselves to potential hazards^26^. Therefore, elderly farmers in endemic areas are now a high-risk population. We highly recommend expanding the HFRS vaccination to people older than 60 years to better protect against the disease.

Using elliptic scan statistics, we detected four significant spatio-temporal “hotspots” areas of HFRS that accounted for nearly half of total cases in Shaanxi Province. Hotspots 1 and 2 were located in the HFRS traditional epidemic areas. Both of the areas were characterized by fertile plains with flourishing agricultural productions, and a high abundance of *A. agrarius* rodents. Hotspot 3 and 4 spanned only three months, reflecting the local outbreaks of HFRS in 2012. All the clustered areas should be prioritized for policy-makers to allocate health resources. In addition, the disease has spread steadily north in recent years. Yan’an and Yulin in the northern part has reported indigenous HFRS cases and infected animal reservoirs, which suggests that the area has newly emerged as a HFRS natural focus^27^. Targeted measures, such as disease and reservoir surveillance, seroprevalance investigation, diagnosis and healthcare service training should also be enhanced in this region.

Our BRT ecological modeling revealed that artificial area, cropland, pig and population density, climate conditions (relative humidity, precipitation, and wind speed), and GDP were drivers of the geographic variation of HFRS. These findings might help us to understand the spatially-clustered distribution of human HFRS cases in Shaanxi. These environmental variables, especially the artificial areas and cropland, may provide a suitable habitat for rodent hosts. Counties/districts with high proportions of both artificial area and cropland were commonly associated with the highest risk of HV infection. These areas were usually situated in rural-urban fringes or densely-populated towns, suffering from dramatic landscape changes, poor sanitation, dense mice populations, and a large transient population, which may greatly increase human exposures^7,28^. In addition, it seemed that the relationships between HFRS incidence and population density and GDP also supported our hypothesis. A certain population density was most likely to facilitate human exposures of rodents or their excreta, but the areas with high GDP per capita in urban districts were mostly accompanied by advanced living conditions and safety awareness, protecting humans from HV infection^10^. In general, climate variability is an important predictor of the seasonal fluctuation of HFRS^29^. Our study suggested that climate could influence the geographic distribution of HFRS as well, e.g., temperate areas with high humidity, moderate precipitation and wind speed had higher HFRS incidences than other places. This finding was consistent with previous studies. Yan *et al*., described areas with high incidences of HFRS that often occurred in the temperate zone of China, and Chen *et al*., showed rodent hosts preferred the temperate area^7,30^. Additionally, our analysis identified a positive relationship between HFRS incidence and pig density. Similar conclusions in previous surveys also demonstrated livestock husbandry, such as pig, deer, and chicken farms, played an intermediate role in HV infections by affecting rodent populations^31,32^.

Some limitations of the study should be acknowledged. First, in China, HTNV- and SEOV-related HFRS cases are mainly associated with two distinct rodent species, i.e., *A. agrarius* and *R. norvegicus*. The former usually occurs in fields, while the latter thrives in residential areas. Hence, the epidemiology of HFRS cases should be differentiated by the two species, and the association between HFRS incidence and environmental elements may differ because each rodent species has its own habitats with special ecological attributes. Unfortunately, the reported HFRS cases in China are not distinguished by causative HV. Second, HFRS cases collected from the hospital-based surveillance system only captures patients who sought medical care, while patients with subclinical or mild infection may be missed because those cases did not seek treatment. Patient underreporting seems unavoidable in similar studies. Third, our model evaluated the relationship between the predictors and HFRS incidence, but the complete transmission mechanism of HV is likely more complicated. Other determinants such as the rodent density, the infected rodent population, the contact frequency between infected rodents and humans, population immunity, and public health initiatives were not included in our analysis because these data were unavailable. These limitations may be addressed in better designed future studies.

## Conclusions

In conclusion, our study provides a precise and detailed picture of the dynamic epidemic patterns of human HFRS in Shaanxi Province, and identified high risk populations, areas, and underlying causes for disease transmission. As evidenced in our statistical analyses, targeted strategies should be formulated and implemented to reduce future local disease incidences.

## Materials and Methods

### Data collection and management

In China, HFRS is listed as a class B notifiable infectious disease, and cases diagnosed at medical institutions are required to be reported to CCDC through the national Notifiable Infectious Diseases Reporting Information System^33^. The case definition of HFRS is according to the guidelines of the World Health Organization (WHO). Briefly, two recommended methods, optic microscopy and rapid diagnostic tests based on lateral flow immunochromatography, are used to identify the clinical symptoms of HFRS^6^. In this study, data regarding human cases of HFRS from January 1, 2005 to December 31, 2016 were collected from the Center for Disease Control and Prevention of the Shaanxi Province. HFRS case information on sex, age, occupation, residence address, onset date of symptoms, hospital admission date, and clinical outcomes were obtained. All records were anonymized prior to analysis. Demographic data from 2005 to 2016 for each county were collected from the National Bureau of Statistics of China.

Based on the published scientific evidence and expert opinions, factors probably associated with HFRS transmission were collected for statistical analysis, including climatic, ecological, and social-economic variables. Climatic data (including temperature, precipitation, relative humidity, wind speed, sunshine hours, and pressure) covering 35 surveillance stations in Shaanxi Province during the study period were downloaded from the China Meteorological Data Sharing Service System (available at http://data.cma.cn). The data were spatially interpolated to a 1 km^2^ resolution grid using a kriging model, and the average value for each county was extracted using ArcGIS 9.3 software (ESRI Inc., Redlands, CA, USA). Land cover data with 300 m resolution were derived from a raster version of “GlobCover 2009 land cover map” (available at http://due.esrin.esa.int/globcover)^34^, which was processed by the European Space Agency. Land cover types were classified as follows: artificial area, irrigated cropland, rainfed cropland, forest, shrubland, orchard, or bare area. Raster-typed density data regarding pig, cattle, and goat were collected from the Food and Agriculture Organization of the United Nations (available at http://www.fao.org/ AG/againfo/resources/en/glw/GLW_dens.html). Data on elevation and gross domestic product (GDP) were collected from the Data Sharing Infrastructure of Earth System Science (available at http://geodata.cn/). The data on soil-selenium were obtained from the Chinese Academy of Agricultural Sciences. All data management and spatial analyses were performed using ArcGIS 9.3 (Table S1).

### Statistical Analysis

#### Description of epidemiological features

A bar chart of weekly and annual incidences was plotted to display the temporal distribution of HFRS. The annual incidence of HFRS for each county was geo-referenced to the county on a digital map of Shaanxi Province to illustrate the spatio-temporal distribution of HFRS. Total human HFRS cases and incidence rates were stratified by sex and age group to evaluate the demographic patterns of the epidemic.

#### Analysis of endemic hotspots

To identify the areas most affected by HFRS, an elliptic scanning window statistic was computed using SaTScan software 9.0 (available at http://www.satscan.org)^35^. We constructed a space-time discrete Poisson model to detect hotspots of HFRS, constraining the maximum spatial size to be ≤15% of the total population and the maximum temporal size to be ≤85% of the study period. The elliptic scan statistic with different axis lengths and angles is better able to explore and identify narrow, long, and noncontiguous areas of high HFRS incidence than the traditional circular window. Statistical significance of the hotspot was determined by Monte Carlo hypothesis testing, which simulated 999 random replications under the null hypothesis to ensure adequate power for identifying significant hotspots. A *P*-value less than 0.05 was considered to be significant. The epidemiological and ecological features were then described for each hotspot, and the areas were marked on the HFRS incidence map.

#### Analysis of risk determinants of HFRS

We built a multivariate boosted regression tree (BRT) model to assess the risk factors related to the geographic heterogeneity of HFRS. BRT modeling is effective for predicting distributions of variables while accounting for non-linear relationships and interactions between covariates, and has been widely used for mapping the occurrence of infectious diseases^26,36–38^. We trained a Poisson regression BRT model using the average incidence from 2005 to 2014 for each county as the outcome and 18 potential factors as co-variables in the analysis. We evaluated several combinations of the learning rate (0.01, 0.03, 0.05), tree complexity (4, 6, 8), and bag fraction (50%, 75%) parameters in the models to identify the most optimal settings, and then the bootstrapping procedure was repeated for 100 iterations on the selected model to obtain a more reliable and stable result. For all co-variables, the BRT relative contributions were calculated, and only those values above the randomness threshold (100%/number of predictors, 100%/18 = 5.56%) were considered to be significant. To better understand the fitted functions, we plotted the curves of partial dependence for each significant predictor on the HFRS incidence. The predictive power of the model was tested using the corresponding predictors in 2015–2016 in the selected model to forecast the geographic distribution of HFRS incidence. The pseudo-*R*^2^ diagnostic was used to assess the model’s predictive power. All statistical analyses were performed using R software (version 3.3.3; R Core Team 2017).

## Electronic supplementary material

Supplementary information
