# Author Correction: Mapping the epidemic changes and risks of hemorrhagic fever with renal syndrome in Shaanxi Province, China, 2005–2016

**DOI:** 10.1038/s41598-018-24102-x

**Published:** 2018-04-13

**Authors:** Weifeng Liang, Xu Gu, Xue Li, Kangjun Zhang, Kejian Wu, Miaomiao Pang, Jianhua Dong, Hunter R. Merrill, Tao Hu, Kun Liu, Zhongjun Shao, Hong Yan

**Affiliations:** 1 Department of Epidemiology and Health Statistics, School of Public Health, Xi’an Jiaotong University College of Medicine, Xi’an, 710061 China; 2 Department of Epidemiology, School of Public Health, Fourth Military Medical University, Xi’an, 710032 China; 3 Department of Epidemiology and Medical Statistics, School of Public Health and Management, Weifang Medical College, Weifang, 261000 China; 4 Department of Mathematics, School of Biomedical Engineering, Fourth Military Medical University, Xi’an, 710032 China; 5 Shaanxi Provincial Corps Hospital of Chinese People’s Armed Police Force, Xi’an, 710054 China; 6 Shaanxi Provincial Center for Disease Control and Prevention, Xi’an, 710054 China; 7 Department of Agricultural and Biological Engineering, University of Florida, Gainesville, Florida 32611 USA; 8 Digital Resources and Information Center, Taishan Medical University, Taian, 271016 China

Correction to: *Scientific Reports* 10.1038/s41598-017-18819-4, published online 15 January 2018

This Article contains typographical errors in the Acknowledgements section.

“This work was supported by the National Natural Science Foundation of China (81172728), the Health Scientific Research Foundation of Shaanxi Province (2006A002), the Natural Science Foundation of Shaanxi Province (2017JQ8015), and the Natural Science Basic Research Plan of the Shaanxi Province (2016JM8088). The funders had no role in study design, data collection and analysis, decision to publish, or preparation of the manuscript.”

should read:

“This work was supported by the National Natural Science Foundation of China (81370082, 81373058, 81773488), the Health Scientific Research Foundation of Shaanxi Province (2016A002), the Natural Science Foundation of Shaanxi Province (2017JQ8015), and the Natural Science Basic Research Plan of the Shaanxi Province (2016JM8088). The funders had no role in study design, data collection and analysis, decision to publish, or preparation of the manuscript.”

