# Supplementary material for: Mapping the epidemic changes and risks of hemorrhagic fever with renal syndrome in Shaanxi Province, China, 2005–2016
Source: Sci Rep. 2018 Jan 15;8:749. doi: 10.1038/s41598-017-18819-4 (PMC5768775; doi:10.1038/s41598-017-18819-4)

Supplementary information:

Mapping the epidemic changes and risks of hemorrhagic fever with renal syndrome in Shaanxi Province, China, 2005-2016

Weifeng Liang[1], Xu Gu[2,3], Xue Li[2], Kangjun Zhang[2], Kejian Wu[4], Miaomiao Pang[5], Jianhua Dong[6], Hunter R. Merrill[7], Tao Hu[8], Kun Liu[2†], Zhongjun Shao[2†], and Hong Yan[1†]

[1] Department of Epidemiology and Health Statistics, School of Public Health, Xi'an Jiaotong University College of Medicine, Xi'an, 710061, China

[2] Department of Epidemiology, School of Public Health, Fourth Military Medical University, Xi'an, 710032, China

[3] Department of Epidemiology and Medical Statistics, School of Public Health and Management, Weifang Medical College, Weifang, 261000, China

[4] Department of Mathematics, School of Biomedical Engineering, Fourth Military Medical University, Xi'an, 710032, China

[5] Shaanxi Provincial Corps Hospital of Chinese People's Armed Police Force, Xi'an, 710054, China

[6] Shaanxi Provincial Center for Disease Control and Prevention, Xi'an, 710054, China

[7] Department of Agricultural and Biological Engineering, University of Florida, Gainesville, Florida, 32611, USA

[8] Digital Resources and Information Center, Taishan Medical University, Taian, 271016, China

[†]Corresponding author: Kun Liu, E-mail: liukun5959@qq.com; Zhongjun Shao, E-mail: 13759981783@163.com; Hong Yan, E-mail: yanhonge@mail.xjtu.edu.cn

Supplementary information

1. **Table S1**
2. **Figure S1-S6**

Table S1. Description of potential influencing factors used in the analyses.

Variables	Description (Unit)	Type
Temperature	Annual average temperature for each county (℃)	Continuous
Precipitation	Annual cumulative precipitation for each county (mm)	Continuous
Relative humidity	Annual average relative humidity for each county (%)	Continuous
Sunshine hours	Annual cumulative sunshine hours during the study period for each county (hours)	Continuous
Wind speed	Annual average wind speed during the study period for each county (m/s)	Continuous
Pressure	Annual average pressure during the study period for each county (hpa)	Continuous
Elevation	Average elevation for each county (m)	Continuous
Percentage coverage of artificial area	Percentage coverage of artificial area for each county (%)	Continuous
Percentage coverage of cropland	Percentage coverage of cropland for each county (%)	Continuous
Percentage coverage of forest	Percentage coverage of forest for each county (%)	Continuous
Percentage coverage of shrub	Percentage coverage of shrub for each county (%)	Continuous
Percentage coverage of orchard	Percentage coverage of orchard for each county (%)	Continuous
GDP	Average gross domestic product for each county (10 million Yuan per km^2)	Continuous
Population density	Population density for each county (1000 persons per county)	Continuous
Pig density	Pig density for each county (head per km^2)	Continuous
Goat density	Goat density for each county (head per km^2)	Continuous
Cattle density	Cattle density for each county (head per km^2)	Continuous
Selenium	Selenium content in each county	Categorical

Figure S1. Annual incidence pyramid of HFRS in Shaanxi Province, 2005-2016.

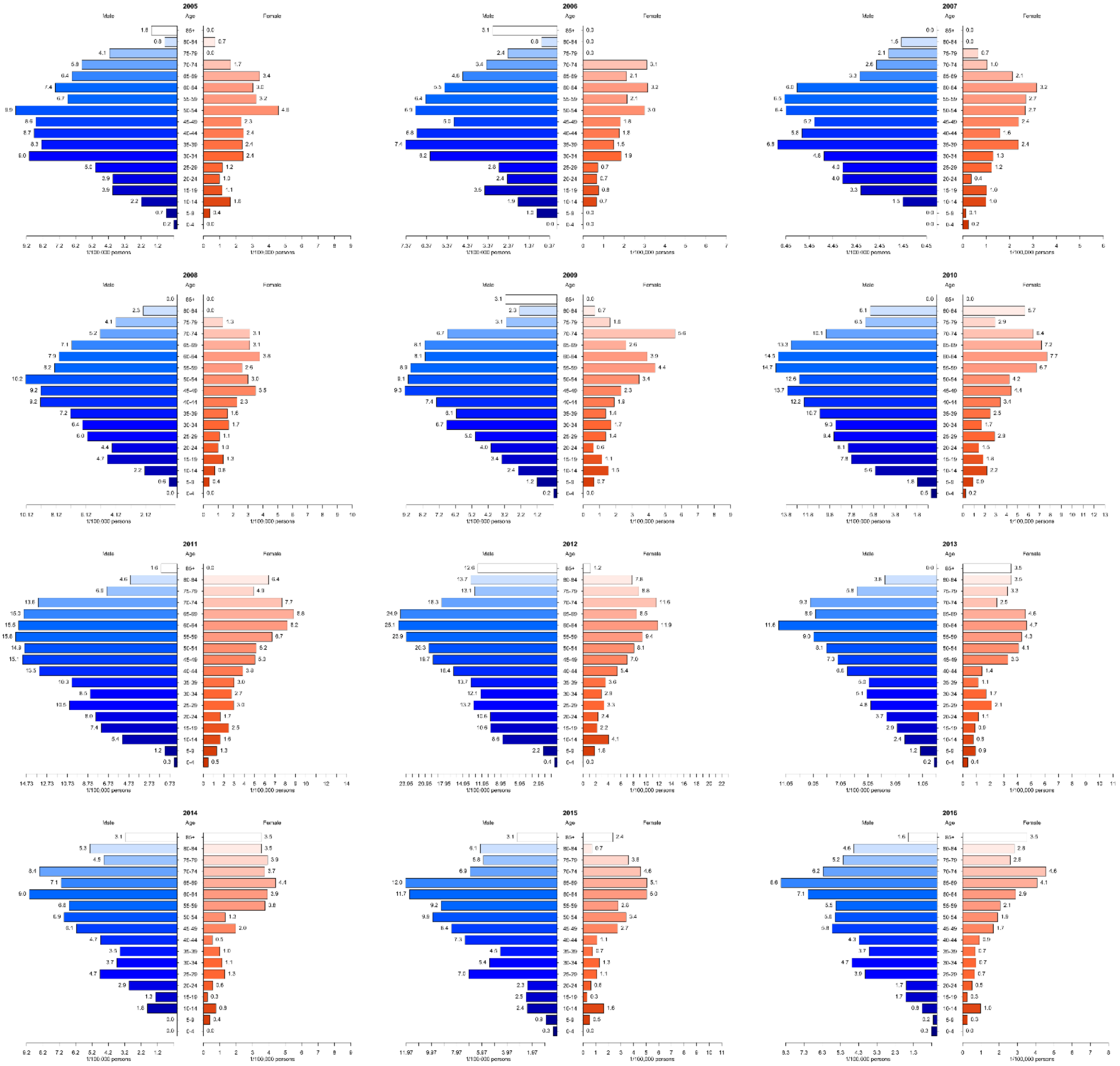

Figure S2. Annual vaccination population in Shaanxi Province from 2003 to 2016.

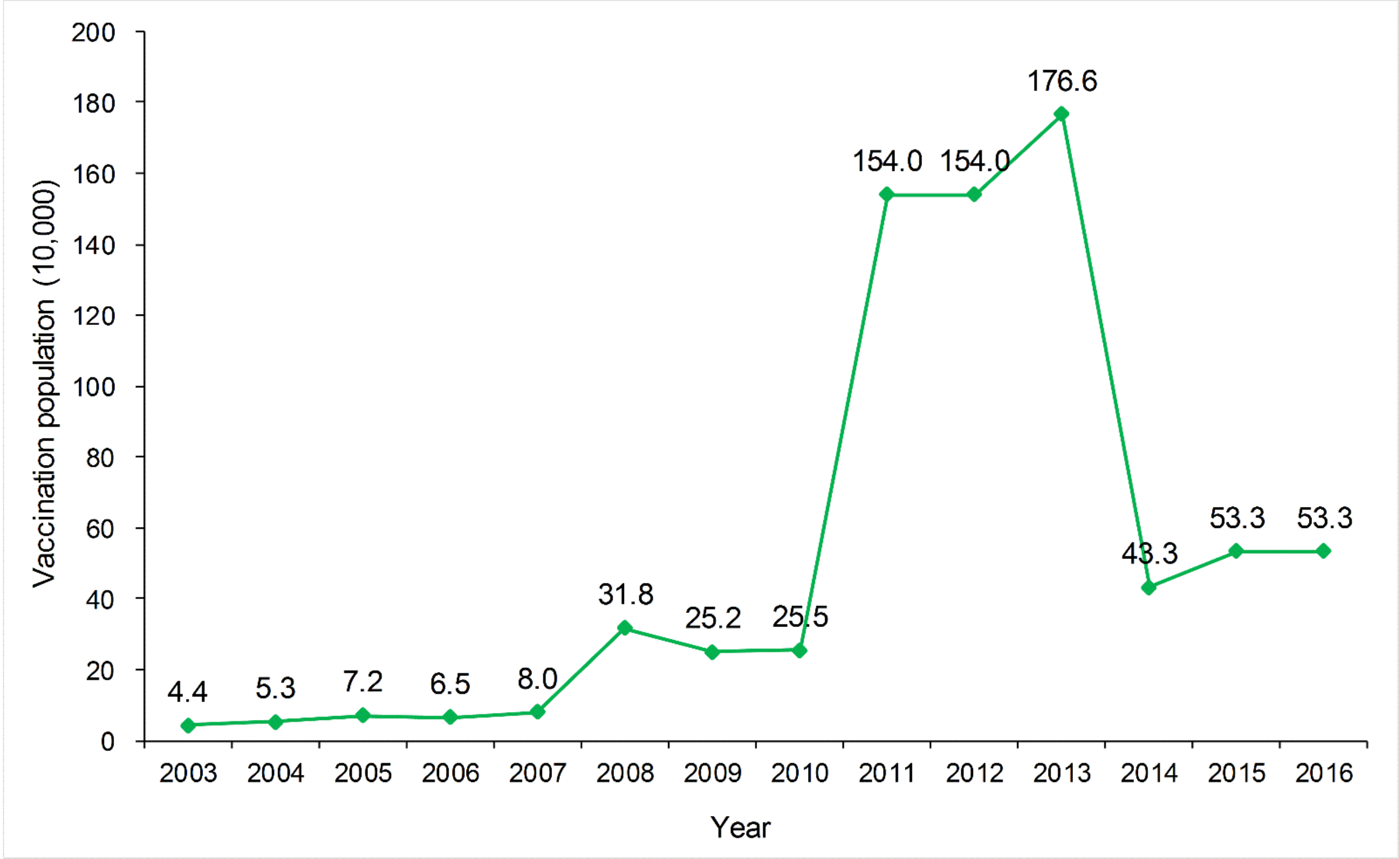

Figure S3. Heatmap of the HFRS weekly average incidences by latitude in Shaanxi Province from January 1, 2005 to December 31, 2016. The average seasonal distribution of HFRS incidences are shown for each latitude, increasing from bottom to top.

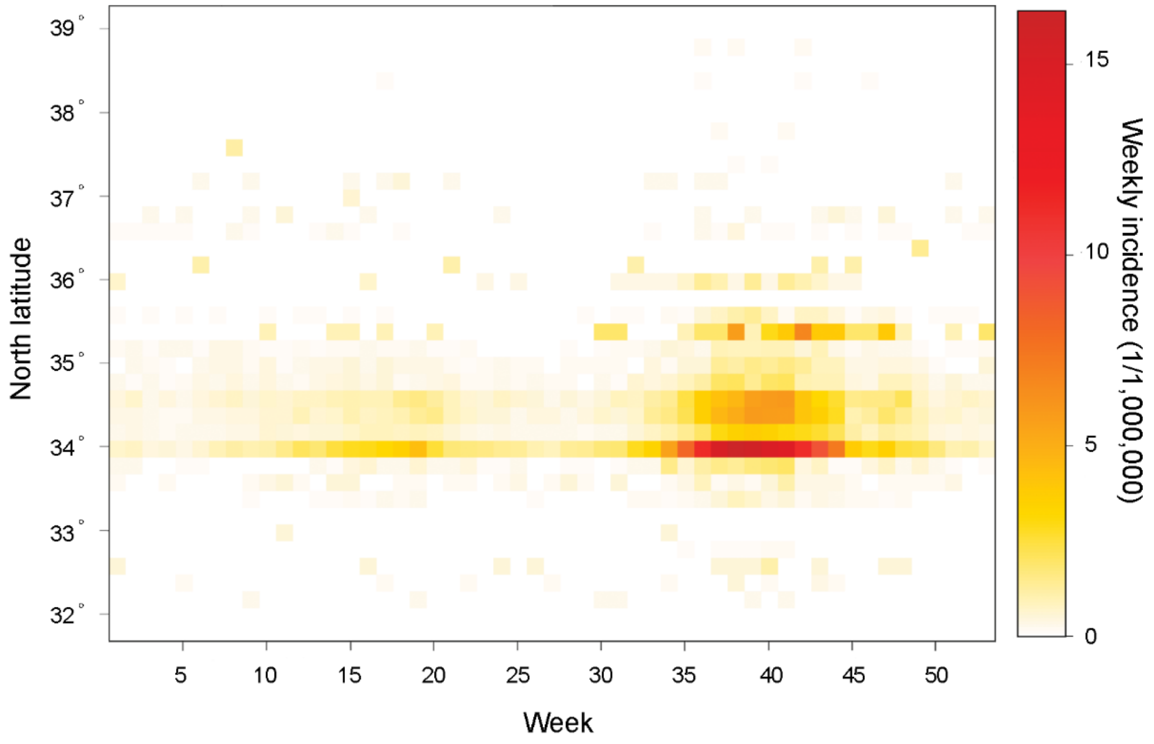

Figure S4. Spatio-temporal hotspots overlapping the annual incidence of HFRS in Shaanxi Province. The map was created in ArcGIS 9.3 software, ESRI Inc., Redlands, CA, USA, (https://www.arcgis.com/features/index.html).

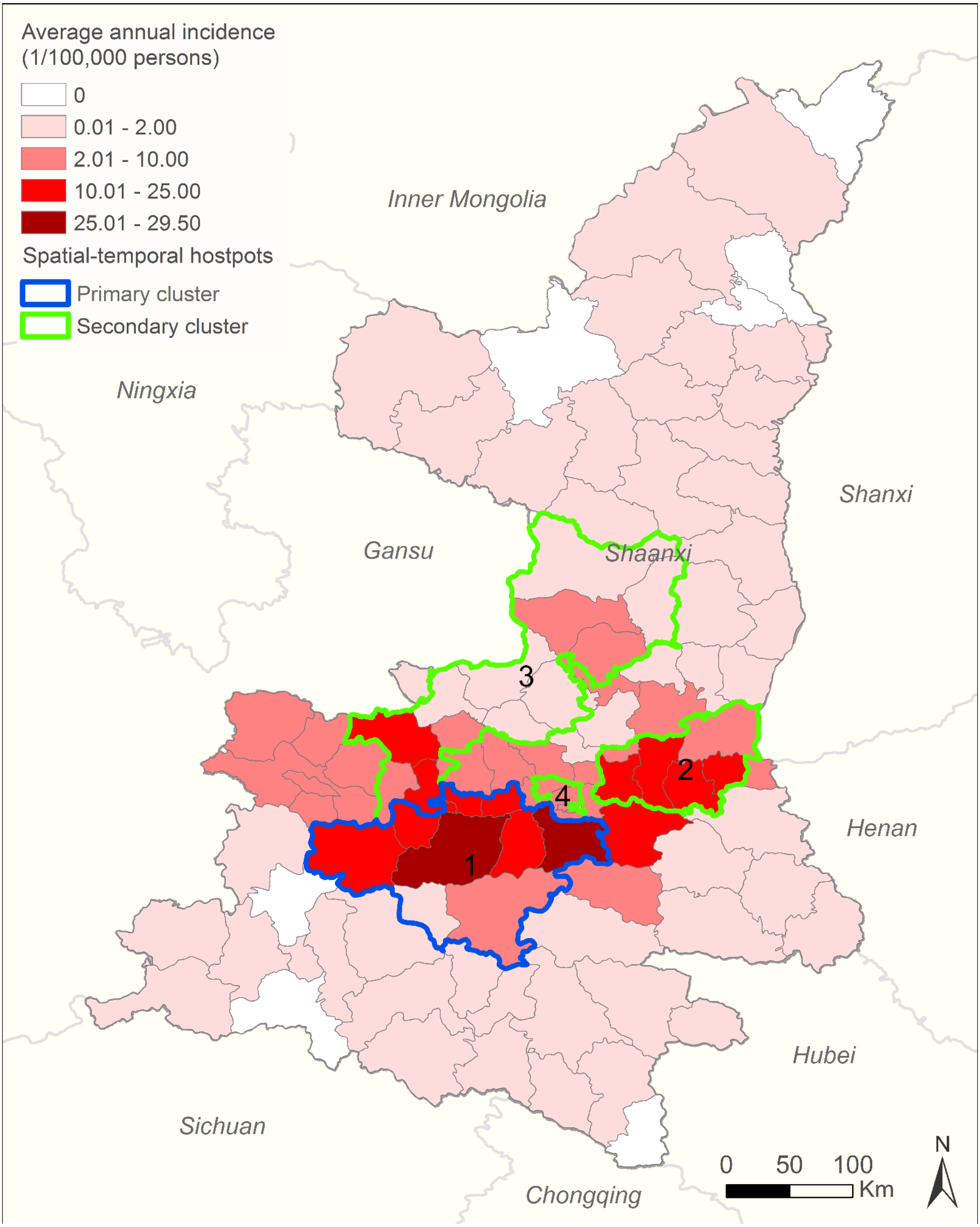

Figure S5. Partial dependence plots showing the effect of different influencing predictors on the HFRS incidence. The black curve indicates the average for the 100 replications and the grey bands represent standard deviation.

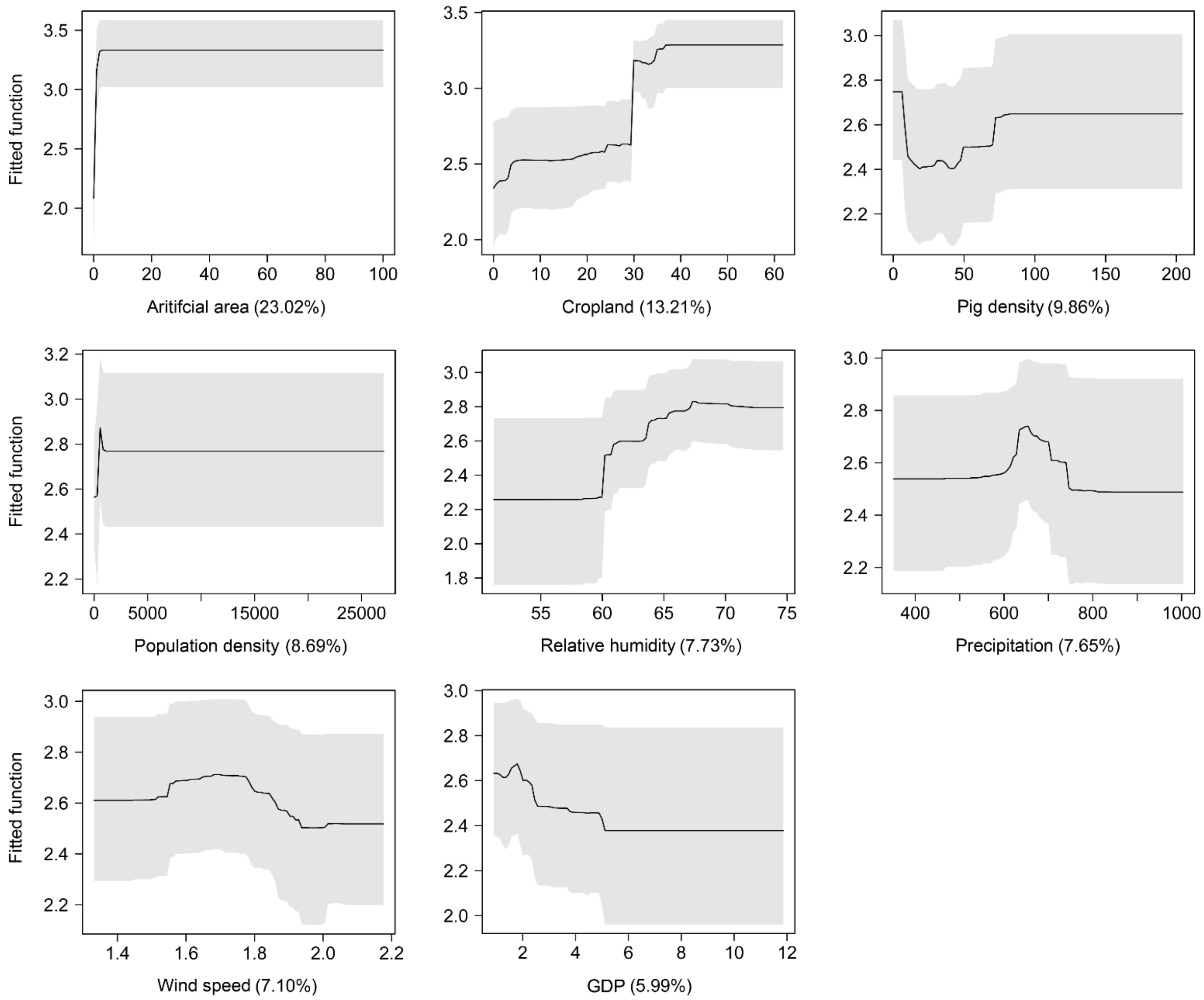

Figure S6. Scatterplot of observed and predicted HFRS incidences at the county level in Shaanxi Province, 2015-2016.

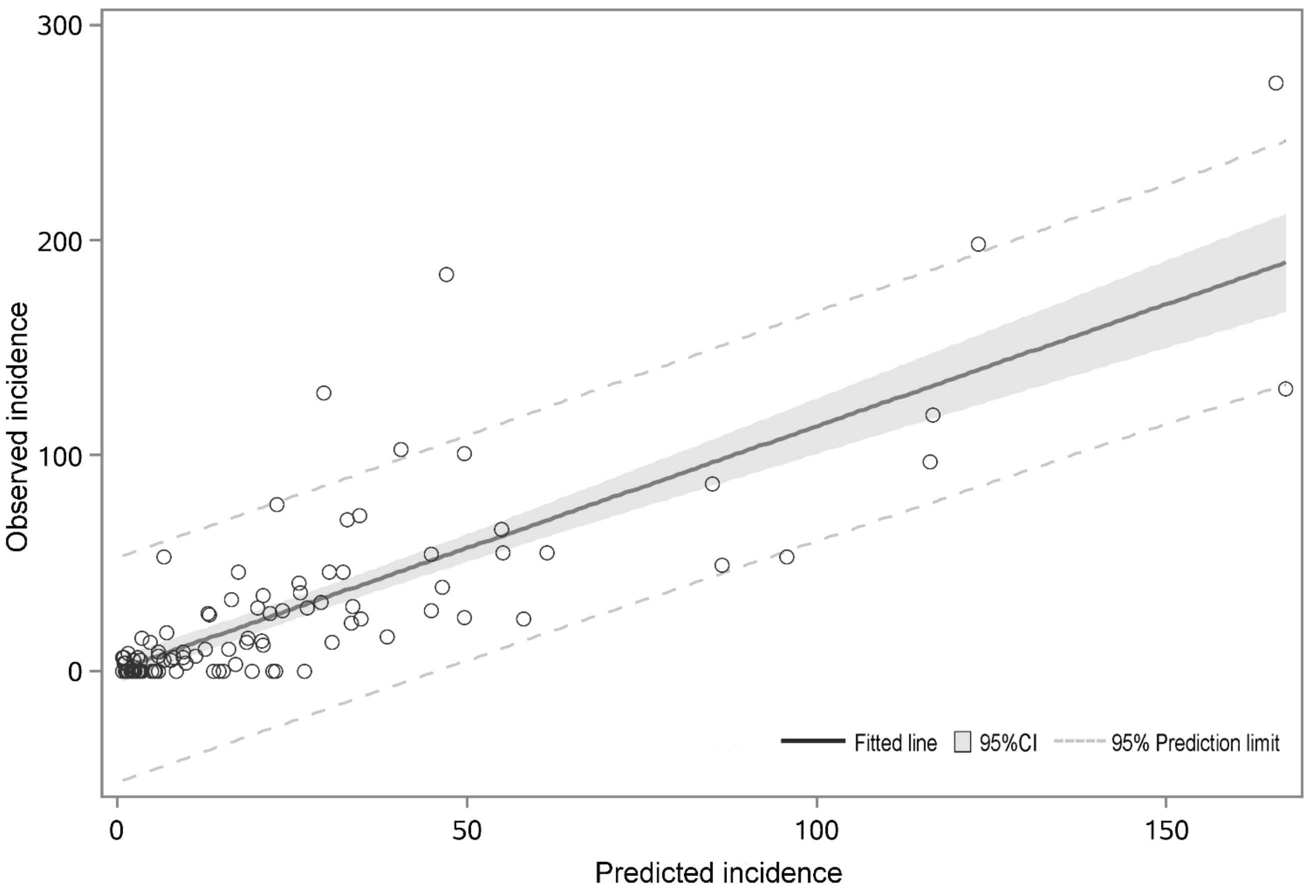

Supplement: Supplementary file 1 — Supplementary information [file 41598_2017_18819_MOESM1_ESM.pdf]
